# Did Expanded Dental Insurance Reduce Out-of-Pocket Expenditures on Dental Care among Older Adults in Korea? Interrupted Time-Series Analysis

**DOI:** 10.3390/ijerph18063003

**Published:** 2021-03-15

**Authors:** Nam-Hee Kim, Se-Hwan Jung, Ichiro Kawachi

**Affiliations:** 1 Department of Dental Hygiene, Wonju College of Medicine, Yonsei University, 20 Ilsan-ro, Wonju 26426, Korea; 2 Department of Social and Behavioral Sciences, Harvard T. H. Chan School of Public Health, 667 Huntington Avenue, Boston, MA 02115, USA; ikawachi@hsph.harvard.edu; 3 Department of Preventive and Public Health Dentistry, College of Dentistry, Gangneung-Wonju National University & Research Institute of Oral Science, Gangneung-Wonju National University, Gangneung 25457, Korea; feeljsh@gwnu.ac.kr

**Keywords:** dental insurance, expenditures, interrupted time-series analysis, counterfactual condition, older adults, causal inference

## Abstract

The Korean National Health Insurance extended its coverage to reduce the economic burden of receiving dentures and implants for older adults in 2012 and 2014, respectively. We examined whether the new policy resulted in reduced out-of-pocket dental care expenditure in the eligible population, specifically focusing on low-income adults. We used interrupted time-series analysis (ITSA), a quasi-experimental design, to identify the effects of the policy among persons aged 65 or older. Data were extracted from the Korea Health Panel Survey (KHP; 2008–2017). The main outcome was out-of-pocket expenditures on dental care. The ITSA showed that expenditures decreased annually by 4.5% (RR: 0.96, 95% CI: 0.95–0.96) between 2012 and 2014. However, expenditure increased by 7.8% (RR: 1.08, 95% CI: 1.07–1.08) after 2014. Dental insurance coverage did not contribute to reducing the out-of-pocket expenses for dentures among low-income adults, while coverage of dental implants led to an increase in dental expenditure.

## 1. Introduction

In many countries, dental care is not included in universal insurance coverage. However, this leads to the social burden of disease that will need to be addressed by health insurance that covers essential oral health care [1,2,3].

The Korean government expanded dental insurance for older adults to cover denture services in 2012, and dental implants in 2014. These steps were expected to reduce the economic barriers to dental care. People aged 65 or older could save approximately 50–70% of their out-of-pocket expenditures for dental prosthetic treatment. However, such treatment may have continued to be unavailable to people with limited financial resources due to high costs [4,5]. For example, the average monthly income of older adults in Korea is less than 1000 USD [6]; however, they still need to pay approximately 700 USD for a covered dental prosthetic treatment (per denture service or a dental implant), regardless of their income level.

In contrast to medical insurance, there is a lack of evidence regarding the effects of dental insurance policies. It is possible that dental insurance has failed to ensure dental care for all due to the late initiation of the policy, its limited coverage, and the remaining out-of-pocket expenditures. Dental insurance will likely remain a low priority unless rigorous evidence is available regarding its social impact.

Previous studies have suggested that dental care reforms in some countries have been accompanied by increasing or persistent income-based inequalities in oral health [5,7,8,9,10,11,12]. This paradox could be due to high socioeconomic status (SES) people using their resources (i.e., money, knowledge, and networks) to capture proportionally more of the benefits of dental care reforms, where eligibility was based purely on an age cutoff (65 years or older), but not on income [11,13,14]. Second, ignoring pre-existing needs with respect to the distribution of risk factors may lead to interventions that are ineffective because people with the greatest need could not afford to access such benefits [9,10,11,15]. For example, there is a direct relationship between income and oral health; that is, the lower one’s SES, the less likely it is that one has access to dental care, even though the need for care is greater [5,7,16]. Furthermore, people with low SES, and specifically older people, might not pursue oral health as a priority on which they are willing to spend their resources [8,17]. In turn, people with high SES spend more on dental care than do people with low SES because dental care is regarded as a “luxury service” and “selective expenditure” that is purchased only when it does not compromise the family budget, resulting in an income gap in dental expenditures [7].

Given these issues, we evaluated whether the expansion of dental insurance for older adults to cover denture and dental implant services (in 2012 and 2014, respectively) led to a reduction in dental care expenditures, focusing on people with low incomes in South Korea.

## 2. Materials and Methods

### 2.1. Study Design and Data Resource

We applied an interrupted time-series, quasi-experimental study design to analyze repeated cross sections within the Korea Health Panel Survey (KHP) data from 2008 to 2017. We performed pre- and post-intervention comparisons, where the interventions were defined as the 2012 policy change (when dentures became subsidized) and the 2014 policy change (when dental implants became subsidized). The KHP is a nationally representative longitudinal survey administered by the Korea National Health Insurance Service and the Korea Institute for Health and Social Affairs. A two-stage stratified cluster sampling design was employed. The survey was conducted based on computer-assisted personal interviews. A detailed description of the KHP methodology is provided elsewhere [18,19,20]. This study focused only on those reporting dental expenditures.

### 2.2. Study Population and Main Variable

The study population consisted of persons aged 65 years or older who were eligible for the dental insurance benefit (mean age: 73.4 ± 6.1 years, range: 65–104). We included approximately 3380 participants who have had dental visits each year. We excluded 428 (0.2%) responses with missing household income values. The sample selection flowchart is shown in Figure S1.

The main outcome, out-of-pocket (OOP) expenditures on dental care, was assessed using self-reported costs for a given year. We considered the OOP expenses for dental care regardless of treatment content and frequency, including pre-paid expenses. In our stratified analyses, we considered people in the top quintile vs. the bottom quintile of incomes.

### 2.3. Statistical Analysis

First, we employed generalized linear model (GLM) regression to estimate the dental expenditures. This was done to remove biases in the predicted means and to control health expenditure data issues; the data have a skewed distribution to the right due to extremely high costs [21,22].

We performed two interrupted time-series analyses: a single- and multi-group interrupted time-series analysis (ITSA). First, segmented regression with an adjusted Poisson model was used for the single-group analysis that included only persons aged 65 or older [23,24].

The following segmented regression model was used:

(1) Yt= β0+ β1T+ β2Xt+ β3TXt

where β0 represents the baseline level of out-of-pocket expenditure at T = 0 (2008); β1 is interpreted as the change in expenditures on dental care, reflecting the annual pre-intervention trend; β2 is the change in the expenditure level following the policy; and β3 indicates the slope of change following the policy using the interaction between year and the policy: TXt.

Second, ordinary least-squares (OLS) regression models, designed to adjust for autocorrelation, were employed for the multi-group ITSA, which included the group with the lowest quintile of income as the treatment group and group with the highest quintile of income as the control group. We employed *newey*, which estimates the coefficients to address autocorrelation and possible heteroscedasticity. Post-estimation time-series analysis to verify the post-intervention trend was implemented using the “itsa” Stata command specifically designed for time-series data [25]. The multi-group analysis is expressed as follows:

(2) Yt= β0+ β1Tt+ β2Xt+ β3XtTt+ β4Z+ β5ZTt+ β6ZXt+ β7ZXtTt+ t

Here, Yt is the expenditure on dental care measured for each year t; Tt is the number of years since the start of the study; Xt is a dummy variable representing the policy (pre-intervention: 0, post-intervention 1); Z is a dummy variable that denotes group assignment (1 = lowest income group, 0 = highest income group); and XtTt, ZTt, ZXt, and ZXtTt are all interaction terms involving the previously described variables.

The coefficients of β0 to β3 represent the control group, and the coefficients of β4 to β7 represent the treatment group. More specifically, β4 represents the difference in the level (intercept) of dental expenditures between the treatment and control groups prior to the intervention; β5 represents the difference in expenditures on the dental care slope (trend) between the treatment and control groups prior to the intervention; β6 indicates the difference in expenditures on dental care between the treatment group and control group immediately following the intervention introduction; and β7 represents the difference in expenditures on dental care slope (trend) between the treatment and control after the policy initiation compared with the pre-intervention slope [25].

Finally, we conducted sensitivity tests to verify the robustness of the ITSA results. These included a placebo intervention time period of 2011 as the base year in both single- and multi-group ITSA. We tested for autocorrelation to ensure a model fit that accounted for the correct autocorrelation structure in the error distribution, using a Cumby–Huizinga general test.

We used Stata statistical software (Stata Corp. 2017. Stata Statistical Software: Release 15. Stata Corp LLC, College Station, TX, USA) for all statistical analyses.

### 2.4. Ethics Approval and Consent to Participate

All participants provided informed consent before responding to the KHP survey. Secondary data from the KHP are publicly available (https://www.khp.re.kr:444/web/data/data.do, accessed on 10 November 2020). Our institute determined that the use of these datasets did not meet the criteria for human subject research and was therefore exempt from Institutional Review Board approval. We confirmed that all methods met the relevant guidelines and regulations.

## 3. Results

There was an increasing trend in OOP expenditures for dental care in those aged 65 years or older from 2008 to 2017. However, there was heterogeneity in the trend according to income. OOP expenditures increased in people with the lowest income but decreased in people with the highest income. In addition, the dental expenditures of older adults with the highest income were much greater and fluctuated more than did those of the lowest income group (Table 1, Figure S2a–d).

**Figure 1:**
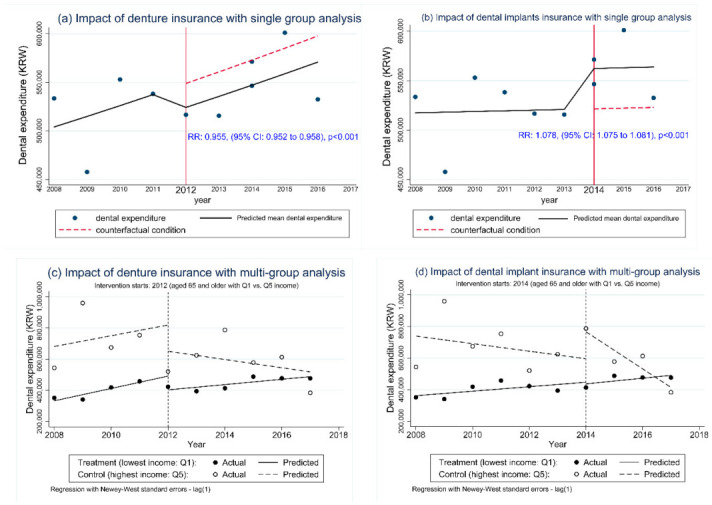
Single- and multi-group interrupted time-series analysis of dental expenditures. (KRW denotes South Korean won).

Expenditures for dental care of older adults decreased after the 2012 policy; in contrast, expenditures increased after the 2014 policy relative to the counterfactual trend in the single-group ITSA model (Figure 1a,b).

We found a level change in the ITSA regression model for dental care expenditures in those aged 65 and over, after both the 2012 and 2014 policy. This implies that there is strong evidence of a downturn in expenditures in the eligible group following the 2012 intervention, with a decline of 4.5% (relative risk (RR): 0.955, 95% CI: 0.952 to 0.958, *p* < 0.001), as illustrated in Figure 1a. Meanwhile, there was an upward trend in expenditures in the same group following the 2014 policy, with an increase of 7.8% (RR: 1.078, 95% CI: 1.075 to 1.081, *p* < 0.001; Figure 1b).

In the multi-group ITSA model, we found that annual expenditures on dental care significantly increased by 97,155 KRW in those aged 65 years or older with the lowest income following the 2014 intervention (β7: 95% CI: 7028 to187,283, *p* < 0.05). By contrast, there was no insurance effect for the lowest income group following the 2012 policy (Figure 1c,d).

Table 2 and Figure 1c show that there was no difference in the pre-intervention expenditures trend between the low vs. high income groups before 2012 (β5: *p* = 0.92). There was a significant drop in OOP expenditures for the low-income group comparing the pre- versus post-2012 policy change (β3 + β7): −22,647 KRW, 95% CI: −42,329 to −2964, *p* < 0.05. There was no significant change in OOP expenses for the high-income group as a result of the 2012 policy (β3 = −60,862, *p* = 0.37).

In the years following the 2012 policy, annual OOP expenses for the low-income group continued to increase (β1 + β3 + β5 + β7): 17,000 KRW, 95% CI: 6987 to 27,000, *p* < 0.001). There was no significant difference in the pre- vs. post-policy trends in OOP expenses comparing the low- and high-income groups (β7: *p* = 0.57).

Figure 1d shows the pre- vs. post-2014 trends in OOP expenditures for the low- and high-income groups. The pre-intervention levels and trends in expenditures were similar for both the low- and high-income groups (β1, β5 + β1, and β5, respectively: *p* > 0.05). After the 2014 policy change, there was a significant decrease of −117,000 KRW in annual expenditures for the high-income group (β1 + β3: 95% CI: −155,000 to −79,800, *p* < 0.001), and the difference between the post- versus pre-intervention expenditures was statistically significant (*p* < 0.05); that is, annual OOP dental expenses among people with the highest income declined to 93,397 KRW after the provision of dental implant coverage. By contrast, there was a significant increase (135,000 KW) in dental care expenses in the lowest income group (β5 + β7: 95% CI: 92,700 to 178,000, *p* < 0.001).

**Figure 2:**
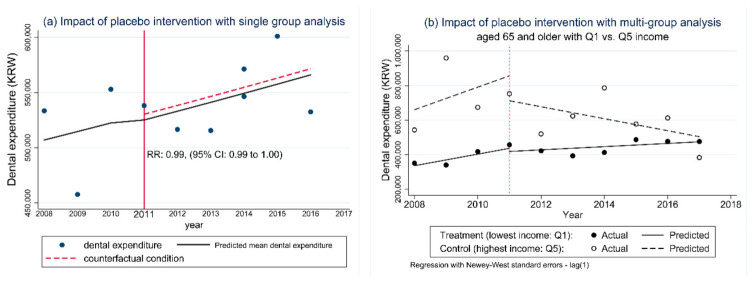
Sensitivity test for the interrupted time-series analysis model. (KRW denotes South Korean won)

We validated our single- and multi-group ITSA models, confirming that there were no significant insurance effects with placebo intervention time in both models (Figure 2 and Table S1). We confirmed the model fit, as evidenced by the significance of the lag (1) of the ITSA models (Table S2). Thus, we used the lag (1) models in the multi-group ITSA to adjust for autocorrelation in 2012 and 2014.

## 4. Discussion

This study used ITSA, a quasi-experimental design, to evaluate whether the 2012 and 2014 policies to reduce the economic barriers to receiving dentures and dental implants actually resulted in reduced OOP expenditures for Koreans aged over 65 years.

The major findings can be summarized as follows. First, the 2012 expanded coverage for denture services had different effects on OOP expenditures compared to the 2014 expansion of coverage for dental implants. Expanded dental insurance for denture services led to reduced out-of-pocket expenditures for dental care. By contrast, coverage of dental implants increased the out-of-pocket expenditure among older adults. Second, an unintended consequence was found. Insurance of dental implants led to increased dental care expenditures among low-income older adults, whereas the reverse was true for high-income older adults.

We employed repeated cross-sections, despite longitudinal panel data, because, in general, older adults have binge visits to dental clinics only when they have trouble. ITSA uses multiple consecutive pre- and post-intervention observations in a single population and incorporates time values. If the counterfactual is estimated by extrapolation of the pre-intervention observations, the trend remains constant [24,26,27].

Our findings have two implications for dental insurance policies directed toward older adults. First, expanding insurance with the objective of reducing OOP expenditures needs to be better targeted. Using age as the only eligibility criterion may be more popular politically, but it is not sufficient to reduce oral health inequities.

Second, an unintended effect of the policy on dental implant coverage appears to have been that it contributes to greater health expenditure among older adults with low income. As such, the government should consider a need-based strategy to achieve oral health equity. Dental treatment is associated with high direct costs as well as indirect loss of income and productivity associated with receiving services [1,28].

Our finding of reduced dental expenditure did not incorporate lag effects [29]. Older adults may have delayed dental treatment, with the expectation of future expansion of benefit eligibility. For example, the OOP percentage was steadily reduced from 50% to 30% during the same period as the age of eligibility was lowered from 75 years or older down to 65 years or older [4].

Contrary to our expectations, dental implant coverage led to increased dental expenses (approximately 100 USD) in the lowest income group. Furthermore, people with the lowest income had greater expenditures (approximately 140 USD) than did people with the highest income after the 2014 policy (Table 2 and Figure 2b). In general, people with higher incomes tend to spend more on dental care [7,30,31,32].

Why did people with the lowest income have the highest dental expenditures, despite being insured? It is possible that older adults with low income are more willing to pay for services partially covered by insurance compared to before the policy change. However, people who have delayed treatment are also more likely present to the dentist with advanced disease status, which might require more elaborate procedures for pre-prosthetic treatment [1,33,34,35].

Some limitations of this study should be addressed. First, self-reported interview data are subject to various biases. Individuals may answer questions incorrectly due to recall error or they may choose to skip a question they feel uncomfortable answering (i.e., income). Second, it was not possible to address cases where an adult child (who did not live with their parents) paid for their parent’s dental expenses. Third, these findings may be applicable only to the South Korean population.

Whether dental insurance has reduced the overall household burden of medical expenses as well as dental expenses remains a subject for further research.

Nevertheless, we investigated the population-level impact of insurance coverage in the Korean context. It is the first study to examine the impact of dental insurance expansion on changes in out-of-pocket expenditures. Although the causal effect of insurance expansion on access/utilization of care has been previously studied, this is the first study to focus on OOP expenditures. Results from our ITSA models provide insight for policymakers regarding the potential health equity impacts of a universal dental coverage scheme on dental care expenditures among older persons. Most importantly, our findings point to an unintended contra-poor consequence of dental insurance policy. Namely, while the policy may have reduced age-related inequity in access to dental care, it worsened income-based inequity in out-of-pocket expenditure.

## 5. Conclusions

Dental insurance coverage for denture service did not contribute to decreased dental expenditures for adults with low income. Dental insurance coverage for dental implants led to an increase in the OOP dental expenditures of the eligible persons with the lowest income.

## Figures and Tables

**Table 1:** Number of participants and expenditure on dental care from 2008 to 2017.

	Aged 65 and Older ^b^				Income Quintile							
					Lowest Income (1st Quintile)				Highest Income (5th Quintile)			
Year	*n*	Mean ^a^	95% CI ^c^		*n*	Mean	95% CI		*n*	Mean	95% CI	
			Lower	Upper			Lower	Upper			Lower	Upper
2008	2826	533,419	396,727	670,111	1159	351,243	229,066	473,420	199	543,440	265,229	821,651
2009	2829	457,749	356,623	558,876	1112	340,571	231,185	449,957	218	959,568	243,232	1,675,905
2010	2876	552,945	451,359	654,531	1127	418,162	259,423	576,900	232	674,084	395,857	952,311
2011	2967	538,179	440,376	635,982	1181	457,466	299,049	615,884	201	753,351	331,709	1,174,994
2012	2979	516,566	440,857	592,276	1168	422,420	322,145	522,696	200	520,553	291,492	749,615
2013	3003	515,598	444,563	586,633	1128	393,791	289,891	497,692	206	623,622	405,143	842,100
2014	4061	546,371	454,968	637,774	1665	413,100	333,199	493,001	270	786,488	52,650	1,520,326
2015	4047	571,270	496,892	645,648	1704	487,369	400,756	573,981	277	577,704	405,373	750,035
2016	4052	600,972	526,261	675,684	1620	477,076	395,055	559,097	284	612,729	424,484	800,974
2017	4158	532,489	476,690	588,288	1650	476,449	396,838	556,060	264	383,789	219,802	547,777

^a^ The dental expenditure unit is KRW. ^b^ The participants who have had dental visits each year whose average age and standard deviation is 73.4 ± 6.1 year old, with a minimal and maximal range from 65 to 104 years old. ^c^ CI denotes confidence interval.

**Table 2:** Lincom estimates for the multi-group comparison.

Measure of Interest	Model Parameter	2012 ^b^			2014 ^c^		
		Point Estimate	*p*-Value	95% CI	Point Estimate	*p*-Value	95% CI
Within-group comparison ^a^							
Pre-intervention trend: control	β1	34,426	0.53	−81,194 to 150,044	−23,911	0.50	−98,843 to 51,022
Pre-intervention trend: treatment	β5 + β1	39,626	0.001	23,939 to 55,313	142,17	0.13	−4684 to 33,117
Difference pre-intervention: treatment versus control	β5	5201	0.92	−111,477 to 121,880	38,127	0.30	−39,152 to 115,406
Difference immediately following the intervention: treatment versus control	β6	80,095	0.61	−249,356 to 409,546	−181,221	0.11	−412,852 to 50,409
Post-intervention trend: control	β1 + β3	−26,400	0.44	−98,800 to 46,000	−117,000	0.001	−155,000 to −79,800
Post-intervention trend: treatment	β1 + β3 + β5 + β7	17,000	0.001	6987 to 27,000	18,000	0.08	−2220 to 38,200
Difference post-intervention: treatment versus control	β5 + β7	43,400	0.22	−2.9700 to 117,000	135,000	0.001	92,700 to 178,000
Difference pre- versus post-intervention: control	β3	−60,862	0.37	−202,176 to 80,453	−93,397	0.03	−177,347 to −9447
Difference pre- versus post-intervention: treatment	β3 + β7	−22,647	0.03	−42,329 to −2964	3759	0.81	−29,035 to 36,552
Difference pre- versus post-intervention: treatment versus control	β7	38,215	0.57	−104,464 to 180,893	97,155	0.04	7028 to 187,283

^a^ Aged 65 years and older with lowest income (treatment) vs. highest income (control). ^b^ Pre-intervention vs. post-intervention for the 2012 policy. ^c^ Pre-intervention vs. post-intervention for the 2014 policy.

## Data Availability

The data that support the finding of this study are available on request from the Korean Health Panel Study (https://www.khp.re.kr (accessed on 10 November 2020)).

## Supplementary Materials

The following are available online at https://www.mdpi.com/1660-4601/18/6/3003/s1, Figure S1: Sample selection flow chart, Figure S2: Dental expenditure for older adults in South Korea, 2008–2018, Table S1: Lincom estimates for the multi-group comparison. Table S2. Cumby-Huizinga test for autocorrelation.
