# Supplementary material for: Did Expanded Dental Insurance Reduce Out-of-Pocket Expenditures on Dental Care among Older Adults in Korea? Interrupted Time-Series Analysis
Source: Int J Environ Res Public Health. 2021 Mar 15;18(6):3003. doi: 10.3390/ijerph18063003 (PMC7999161; doi:10.3390/ijerph18063003)
Supplement: Supplementary file 1 [file ijerph-18-03003-s001.pdf]

Supplementary materials

Figure S1. Sample selection flow chart.

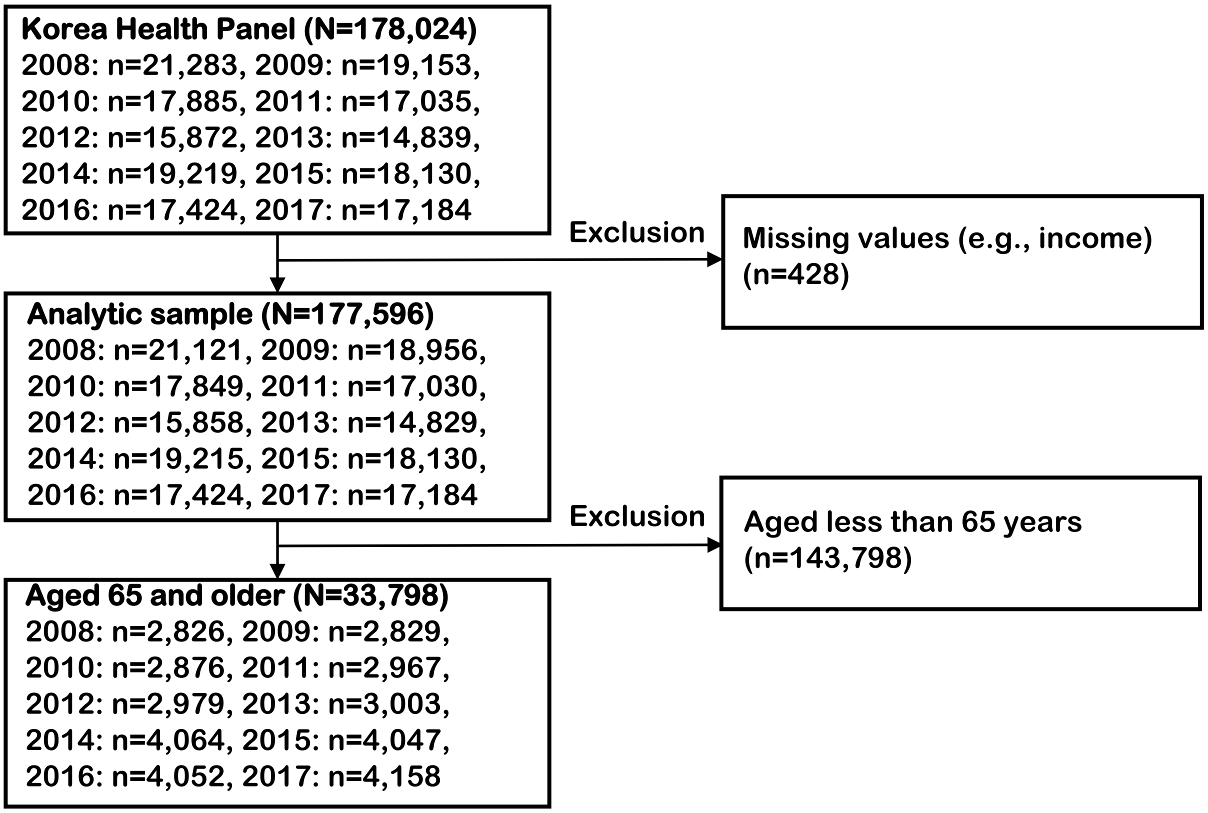

Figure S2. Dental expenditure for older adults in South Korea, 2008-2018

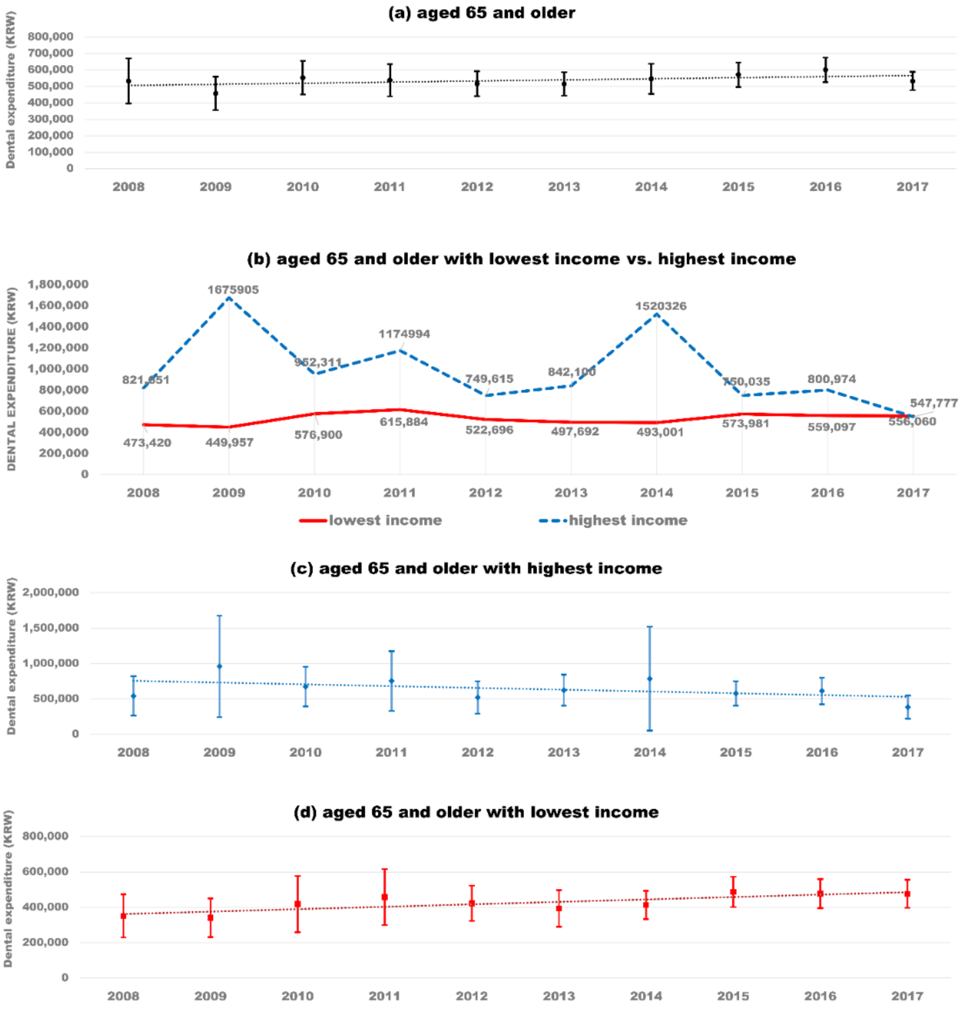

Table S1. Lincom estimates for the multi-group comparison.

Measure of interest	Model parameter	2011 [b]		
		Point estimate	P-value	95% CI
**Between-group comparison [a]**				
Pre-intervention trend: control	β1	65,322	0.55	-167,259 to 297,903
Pre-intervention trend: treatment	β5 + β1	33,460	0.03	4,202 to 62,718
Difference pre-intervention: treatment versus control	β5	-31,863	0.77	-266,277 to 202,552
Difference immediately following the intervention: treatment versus control	β6	126,126	0.62	-419,535 to 671,786
Post-intervention trend: control	β1 + β3	-34,700	0.10	-77,400 to 8,143
Post-intervention trend: treatment	β1 + β3 + β5 + β7	9,280	0.15	-3,800 to 22,400
Difference post-intervention: treatment versus control	β5 + β7	43,900	0.05	-819 to 88,700
Difference pre- versus post-intervention: control	β3	-99,974	0.37	332,115 to 132,168
Difference pre- versus post-intervention: treatment	β3 + β7	24,180	0.17	59,779 to 11,420
Difference pre- versus post-intervention: treatment versus control	β7	75,794	0.50	-159,061 to 310,650

[a] aged 65 years and older with lowest income (treatment) vs. highest income (control).

[b] pre-intervention vs. post-intervention with placebo start in 2011 year.

Table S2. Cumby-Huizinga test for autocorrelation.

lags	Chi^2	df	p-value	lag	Ch^2	dh	p-value
**1-1**	**5.199**	**1**	**0.0226**	**1**	**5.199**	**1**	**0.0226**
1-2	6.961	2	0.0308	2	3.665	1	0.0556
1-3	6.962	3	0.0731	3	0.997	1	0.3179
1-4	7.09	4	0.1312	4	0.937	1	0.333

actest, lags (4)

* Eigenvalues adjusted to ensure a positive semidefinite matrix

H0: variable is MA process up to order q

HA: serial correlation present at specified lags >q

Test allows predetermined regressors/instruments

Test requires conditional homoscedasticity

H0: q = 0 (serially uncorrelated) | H0: q = specified lag-1

HA: s.c. present at range specified | HA: s.c. present at lag specified
